# 

**Published:** 1909-12-04

**Authors:** 


### BOOKS RECEIVED.

University Correspondence College.
" Matriculation Directory."
" The London University Guide."

John Wright and Sons, Ltd.
" Diseases of Metabolism and Nutrition. No. VIII. Gout." By Professor D. H. Strauss.

### Appointments.

Dr. Percy Lunn, of Alverstoke, has been appointed hon. anæsthetist at Gosport.

Dr. Frederick W. Price, M.D., M.R.C.P., has been appointed Physician to the Great Northern Central Hospital.

Dr. J. W. Linnell, B.A., M.B., has been appointed resident medical officer to the Mount Vernon Hospital for Consumption, Hampstead.

Dr. Archibald Todrick, M.B., Ch.B., has been appointed house physician to the Mount Vernon Hospital for Consumption, Hampstead.

Mr. Septimus Lee, M.R.C.S.Eng., L.R.C.P. (Lond.), late resident medical officer of the Hospital for Epilepsy and Paralysis, Maida Vale, N., has been appointed assistant medical officer to the Wye House Asylum, Buxton.

Dr. R. J. M. Buchanan, M.D., F.R.C.P. (Lond.), honorary physician to out-patients and senior assistant physician at the Liverpool Royal Infirmary, has been elected full honorary physician to the Royal Infirmary on the retirement of Sir James Barr.

Manchester Royal Infirmary.—The following have been made by the Board of Management: A. Ramsbottom, M.D., M.R.C.P., Assistant Medical Officer (re-appointed). House Physicians, A. A. Smalley, M.B., Ch.B. (Vict.); N. Tattersall, M.B., Ch.B. (Vict.); G. E. E. Nicholls, M.B., Ch.B. (Vict.). Senior House Surgeons, N. Booth, M.B., Ch.B. (Vict.); T. M. Popple, M.B., Ch.B. (Vict.). Junior House Surgeons, G. Jefferson, M.R.C.S., L.R.C.P.; H. Platt, M.B., Ch.B. (Vict.)

### Appointments Vacant.

Huntingdon County Hospital.—House surgeon.
London Homœopathic Hospital.—Physician.
Royal Albert Hospital, Devonport.—Resident medical officer.

### The Question Box.

**SPECIAL NOTICE.**—Questions of interest affecting the honorary medical staff and hospital administration in all departments are invited. When any point arises we hope our readers will formulate it in a question and so co-operate to make this column of real value and interest. In this way the experience of the best-managed hospitals should be made helpful to the whole hospital world. We shall welcome the contribution of both questions and answers.

**N.B.**—The publication of an answer in this column does not necessarily preclude the publication of subsequent answers to the same question, for a question may involve points which require to be threshed out and fully discussed. Answers, if published, will be paid for at scale rates.

### HOSPITAL ATTENDANCES OF HONORARY MEDICAL STAFF.

### Should an Attendance Book be Kept.

Question 1: Is it practicable to keep a book at each hospital in which the attending physicians and surgeons enter their name on arrival and departure?

A Manchester View.

Answer: Yes.

By Mr. W. G. Carnt, General Superintendent and Secretary Manchester Royal Infirmary.

An attendance book for the Honorary Medical Staff is practicable, and a distinct advantage both to the hospital and the staff. It is little or no trouble to the staff. The book should lie upon a table in the hall and on entering the hospital they should sign their names and enter the time of arrival; they should also, on departure, enter the hour of leaving. It creates a permanent record of their attendance of which they have every reason to be proud. I have heard the opinion expressed that if such a book were suggested in hospitals where it does not exist the medical staff would feel the suggestion to be somewhat of an insult, and an attempt on the part of the committee to check their attendance and keep an eye upon their work. I should like to say at once that the committee have every right to ask for a record of their attendance if they so desire. As a rule, the medical staff are expected to be in attendance at fixed hours if they have out-patients to see, and in spite of the fact that in a great many provincial hospitals the members of the staff are general practitioners with many uncertain calls upon their time, they manage to attend at the hospital at the hour specified with surprising regularity and spend long hours in the consulting-room seeing scores of old and new patients. The staff who attend with regularity should be proud to keep such a record of service, and if by any chance there is one unpunctual member, the fact of the regularity of his colleagues being recorded and compared with his own irregularity should spur him on to conquer his failing, which is, as a rule, caused by lack of method rather than by the intention of neglecting his duties. It is not, however, as a check upon the staff that I think this book should be kept. I advocate it for the sake of the record of the immense amount of time devoted by the honorary staff to the service of the sick. Hospital committees and subscribers have very little conception of the huge amount of gratuitous work performed by their medical staffs. The patients enter and leave the hospital. The committee are intensely interested when the statistics are laid before them at their meetings, but the figures showing hundreds or thousands of cases admitted fail to convey to their minds the accumulating hours of service by the staff. An attendance book, giving not only their regular daily visits, but the many emergency calls paid to ascertain the condition of critical cases, and, in the case of surgeons, to perform long and serious operations (frequently in the night), would be a revelation to many governors, who are not fully aware of all the details of the inner administration of a voluntary hospital.

A Newcastle Practice.

Answer: Yes.

By Mr. R. Orde, House Governor and Secretary, Royal Victoria Infirmary, Newcastle-on-Tyne.

At this hospital an attendance-book has been kept for many years by the attending physicians and surgeons, and, on the whole, with satisfactory results.

A Birmingham View.

Answer: No.

By Mr. Howard J. Collins, House Governor, Birmingham General Hospital.

It is very difficult to answer the questions asked, as "circumstances alter cases," and this is especially so with regard to hospital regulations and even titles. "Honorary medical staff" includes quite different officials in different institutions, and in provincial hospitals, generally speaking, "honorary staff" refers only to the senior members, but in my opinion any medical man appointed on the staff of a hospital for a series of years without salary or honorarium cannot be treated in the same way as a junior member of the staff who receives payment and is appointed only for a short term, such as one or three years. The construction of a hospital and the different methods of appointment will also prevent uniformity of practice, but presuming that the "honorary medical staff" means only the senior physicians, surgeons, and specialists, who have beds in the hospital and deal with in-patients, my answer would be:—

(1) No. It is not practicable to keep a book in which the physicians and surgeons enter their names on arrival and departure, for the reasons—

(a) That such a book, if at the entrance, would be passed by a member of the staff driving in to a courtyard, and (b) if in the hospital proper it would be frequently forgotten.

A Leicester View.

Answer: Yes.

By Mr. Harry Johnson, House Governor and Secretary, Leicester Infirmary.

It is practicable to keep a book in which the times of the attending physicians and surgeons are recorded on arrival and departure. Such a record must be a very helpful administrative feature.

### CONSUMPTION SANATORIA.

Dr. J. G. Wood, M.B., Ch.B., writes: "I would be very glad if you could give me information as to whether there are any free sanatoria convenient to the South of England, or at moderate cost within the means of the working-class, with, if possible, some idea as to the manner of obtaining admission."

[There is no special sanatorium of the kind for the working-classes, we believe. Dr. Wood might apply to King Edward VII. Sanatorium for Consumption, Midhurst, Sussex, to the Royal National Hospital for Consumption, Ventnor, and to the Hahnemann Convalescent Home, West Cliff, Bournemouth.—Ed. The Hospital.]

### LADIES' GUILDS AND COMMITTEES.

A Hospital Chairman writes: "Can you tell me where I can get information as to the best lines on which to run ladies' guilds and committees? How can ladies best help a general hospital? Is it feasible for ladies to meet at the hospital once a fortnight and do needlework on the premises?"

[We refer these questions to our readers. We shall welcome any experience on the points raised.—Ed. The Hospital.]

### Gifts and Bequests.

Mr. Enoch Wheeler has left £100 to the Guest Hospital, Dudley.

Mr. Alexander Fraser has left by will £100 to the Royal Hospital for Incurables, Putney Heath.

The Samaritan Free Hospital for Women has received a donation of £21 from the Skinners' Company.

The Middlesex Hospital has received from the Skinners' Company a further grant of 50 guineas to the general fund.

Mr. Arthur John Hone has left by will £100 to the London Hospital and £100 to the Great Northern Hospital, Holloway Road.

The will of the late Mrs. Gertrude Baxendale, of Portman Square and Welwyn, Herts, contains a bequest of £50 to the Welwyn Cottage Hospital.

Queen Charlotte's Lying-in Hospital, Marylebone Road, N.W., has received a grant of £50 from the Corporation of the City of London.

The Hampstead General Hospital, with which is amalgamated the North-West London Hospital, has received a grant of £105 from the Mercers' Company.

The Committee of the Infants Hospital, Vincent Square, Westminster, gratefully acknowledges the receipt of a donation of ten guineas from Mrs. McCalmont.

The Poplar Hospital for Accidents has received a donation of £100 from Sir Edgar Speyer, the President, and a further anonymous donation of the same amount.

Mr. William Charles Jones, M.A., chairman of the Manchester Skin Disease Hospital, who died on October 3, left £10,000 to the Skin Hospital of Manchester and Salford.

Mr. John Rollings has left by will £50 to the Wolverhampton Eye Hospital; £50 to the Women's Hospital, Wolverhampton; and £50 to the Wolverhampton General Hospital.

A donation of 50,000 Fletton bricks, with free cartage, has been received from Mr. A. W. Ker by the committee for the removal of King's College Hospital to South London. The new site will be at Denmark Hill.

The Worshipful Company of Skinners have given a donation of ten guineas to the funds of the After-Care Association for Poor Persons Discharged Recovered from Asylums for the Insane, Church House, Dean's Yard, Westminster, S.W.

Miss Elizabeth Cornewall has left £100 to the Windsor Royal Infirmary, and £50 to Mr. J. C. Mackinlay, desiring, but without imposing any legal obligation, that he will use this towards the maintenance of the beds which he maintains in the female wards of the Royal Free Hospital.

Miss Emily Lucas, of Bristol, who left estate valued at £14,590 gross, bequeathed £200 to the Temporary Home, £200 to the General Hospital, £100 to the Hahnemann Hospital, £50 to the Royal Infirmary, and £25 to the Royal Hospital for Women and Children—all Bristol institutions.

### THE GRADUATES' MEDICAL SCHOOLS.

DIARY FOR THE WEEK, DECEMBER 6 to 11.

### ST. JOHN'S HOSPITAL FOR DISEASES OF THE SKIN, Leicester Square, W.C.

CHESTERFIELD LECTURES.—At the Hospital on Thursday evenings at 6 p.m., each lecture followed by clinical demonstrations :—

**Dec. 9. Fungous Diseases of the Skin**: I., Tinea Circinata; II., Tinea Imbricata; III., Tinea Versicolor; IV., Erythrasma; V., Actinomycosis; VI., Madura Foot.

### NATIONAL HOSPITAL FOR THE PARALYSED AND EPILEPTIC, Queen Sq., Bloomsbury, W.C.

**At 3.30 p.m.—Dec. 7,** Dr. James Collier, **Cerebral Syphilis.**

**At 3.30 p.m.—Dec. 10,** Dr. Aldron Turner, **Muscular Atrophy.**

### NORTH-EAST LONDON POST-GRADUATE COLLEGE, Prince of Wales's General Hospital, Tottenham, N.

**At 4.30 p.m.—Dec. 7,** Dr. A. de Prenderville, **The Status Lymphaticus and some other Anæsthetic Dangers.**

**At 4.30 p.m.—Dec. 9,** Dr. A. J. Whiting, **Exophthalmic Goitre.**

### MEDICAL GRADUATES' COLLEGE AND POLYCLINIC, 22 Chenies Street, W.C.

**At 5.15 p.m.—Dec. 6,** Dr. David Ferrier, **The Ætiology and Treatment of Tabes.**

**At 5.15 p.m.—Dec. 7,** Dr. Percy Kidd, **Some Points in the Clinical History of Pneumonia.**

**At 5.15 p.m.—Dec. 8,** Dr. E. F. Bashford, **Some Results of the Comparative Biological and Experimental Study of Cancer.**

**At 5.15 p.m.—Dec. 9,** Professor W. Thorburn (Manchester), **Some Points in the Surgery of the Biliary Passages.**

### THE HOSPITAL FOR SICK CHILDREN, Great Ormond Street, W.C.

**At 4 p.m.—Dec. 9,** Mr. Lane, **Some Abdominal Conditions.**

### LONDON SCHOOL OF CLINICAL MEDICINE, Seamen's Hospital, Greenwich, S.E.

**At 2.15 p.m.—Dec. 7,** Dr. Russell Wells, **Some Points in Cardiac Pathology.**

**At 2.30 p.m.—Dec. 9,** Dr. Rankin, **The Therapeutic Value of Rest.**

### THE POST-GRADUATE COLLEGE, West London Hospital, Hammersmith, W.

**At 10 a.m.—Dec. 6 and 9,** Surgical Registrar, **Demonstration.**

**At 10 a.m.—Dec. 10,** Medical Registrar, **Demonstration.**

**At 12 noon.—Dec. 9,** Dr. Bernstein, **Pathological Demonstration.**

**At 12.15 p.m.—Dec. 7,** Dr. Pritchard, **Practical Medicine.**

**At 12.15 p.m.—Dec. 8 and 11,** Dr. Grainger Stewart, **Practical Medicine.**

**At 5 p.m.—Dec. 6,** Mr. Baldwin, **Practical Surgery**—V.

**At 5 p.m.—Dec. 7,** Dr. Robert Jones, **The Treatment of Mental Cases by the General Practitioner.**

**At 5 p.m.—Dec. 8,** Dr. Low, **Prophylaxis of Tropical Diseases.**

**At 5 p.m.—Dec. 9,** Mr. Lloyd, **Anæsthetics.**

**At 5 p.m.—Dec. 10,** Dr. Seymour Taylor, **Pneumonia.**

